# Exploring the Prevention of Lipid Deposition Caused by High‐Fat Diet and Its Mechanism of Action of *Rosa roxburghii* Fermented Juice Based on Liver Metabolomics and Gut Microbiota

**DOI:** 10.1002/fsn3.70449

**Published:** 2025-07-28

**Authors:** Duo Meng, Pengjiao Wang, Shuo Zhang, Zhiyu Chen, Chencen Lai, Xinxin Yi, Xuncai Huang, Haoxiang Yu, Min Zhang, Xiuli Gao

**Affiliations:** ^1^ State Key Laboratory of Discovery and Utilization of Functional Components in Traditional Chinese Medicine and school of pharmaceutical science Guizhou Medical University Guiyang China; ^2^ Engineering Research Center of Microbiology and Biochemical Pharmaceutical, Guizhou Provincial Department of Education Guizhou Medical University Guiyang China; ^3^ Experimental Animal Center of Guizhou Medical University Guiyang China; ^4^ Guizhou Provincial Engineering Research Center of Food Nutrition and Health Guizhou Medical University Guiyang China

**Keywords:** gut microbiota, lipid deposition, liver metabolism, *Rosa roxburghii* fermented juice

## Abstract

Hyperlipidemia has become a prevalent disease in the global epidemic, posing a threat to human health. This study aims to investigate the mechanism by which *Rosa roxburghii* fermented juice (RRFJ) can prevent lipid deposition induced by a high‐fat diet in mice. The results showed that mice in the RRFJ intervention group had significantly reduced body weight as well as lower levels of serum and liver lipid indicators compared to the high‐fat diet group. Metagenomic analysis revealed that the RRFJ intervention reversed the decrease in intestinal flora *Alistipes* and *Colidextribacter* genes in mice fed a high‐fat diet. Liver metabolomics showed that the RRFJ prevented liver dyslipidemia by modulating the biosynthesis of phenylalanine, tyrosine, tryptophan, and phenylalanine metabolism. RRFJ is effective in preventing dyslipidemia through the ‘gut‐liver axis’, which regulates the imbalance of intestinal flora and improves hepatic metabolic profiles. This provides a new intervention strategy for the prevention and treatment of hyperlipidemia.

## Graphical abstract

*Rosa roxburghii* fermented juice can improve dyslipidaemia by modulating gut microbiota disorders. *Rosa roxburghii* fermented juice may regulate lipid deposition by increasing metabolites such as choline, L‐Tyrosine, and L‐Phenylalanine in the liver to improve liver metabolic profile. *Rosa roxburghii* fermented juice may be based on the gut‐liver axis, which works together to balance gut microbiota and improve liver metabolic profile, preventing lipid abnormalities.

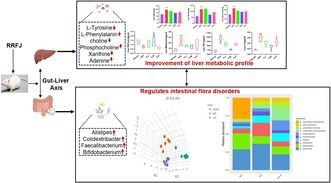

## 1 Introduction

Hyperlipidemia, a common metabolic disease that plays a significant role in the development of cardiovascular diseases and diabetes (Jiang et al. 2021; Mainieri et al. 2023), is closely linked to abnormal blood lipids and lipid deposition, leading to hyperlipidemia, which can directly cause metabolic disorders and pose a serious threat to intestinal health (Chen, Wang, et al. 2016; Guo et al. 2020; Wen et al. 2023). Long‐term use of lipid‐lowering drugs, such as statins, bile acid isolators, niacin, and cholesterol absorption inhibitors, can result in various side effects including muscle pain, gastrointestinal reactions, nausea, and sleep disturbances (Selva‐O'Callaghan et al. 2018; Pisciotta et al. 2015). As a result, the importance of healthy or functional foods in preventing hyperlipidemia and cardiovascular diseases has increased.

*Rosa roxburghii* Tratt (RRT) is a plant that grows in the mountainous regions of southwestern Guizhou, China. It has been traditionally used for both medicinal and edible purposes in China and is recognized as a Guizhou ethnic medicinal material (Ji, Zhang, Yuan, et al. 2022; Yang et al. 2022). RRT is a rich source of nutrients and functional components such as flavonoids, vitamin C, three mushroom compounds, organic acids, polyphenols, and superoxide dismutase (Liu et al. 2016). In recent years, RRT has gained significant attention as a functional food and dietary supplement due to its demonstrated anti‐hyperlipidemic, antioxidant, anti‐atherosclerotic, and anti‐tumor properties (Xu et al. 2019; Su et al. 2022; Song and Shen 2021). Studies have demonstrated that the bioactive components in RRT exhibit multiple pharmacological activities. Specifically, *Rosa roxburghii* polysaccharides have been shown to reduce blood lipid levels in diabetic mice, enhance hepatic antioxidant activity, and improve hyperglycemia and hyperlipidemia in diabetic conditions by modulating gut microbiota, increasing the abundance of beneficial Lactobacillales while decreasing endotoxin‐producing Enterococcaceae and Desulfovibrionaceae (Wang, Li, et al. 2020). *Rosa roxburghii* polyphenols have been found to reduce acute lung injury, modulate immunity, lower blood sugar and lipids, and have anti‐inflammatory effects (Tang et al. 2023, 2022; Wang, Chen, et al. 2022; Liu et al. 2023). Experimental studies have further demonstrated that *Rosa roxburghii* tratt juice can reduce hepatic 8‐OHdG and MDA levels in mice, thereby alleviating liver injury; the hepatoprotective effects are achieved through modulation of nuclear receptor‐mediated pathways, including CAR, PXR, and Nrf2 signaling, which collectively reduce oxidative stress and improve lipid metabolism (Yang et al. 2022). The group's previous studies found that *Rosa roxburghii* tratt juice can regulate the intestinal microbial community and related metabolites, improve lipid absorption, and thus exert a hypolipidemic effect (Ji, Zhang, Tang, et al. 2022). These studies suggest that RRT can exert a hypolipidemic effect by reducing the level of oxidative stress and maintaining intestinal microbial homeostasis in patients with hyperlipidemia through a synergistic multi‐pathway approach.

*Rosa roxburghii* fermented juice (RRFJ) is produced by fermenting organic *rosa roxburghii* fruit juice in a natural environment to preserve active ingredients and maintain a mild flavor. Fresh RRT fruits cannot be stored for long periods at room temperature, so fermentation is used to maximize the preservation of active ingredients and improve taste. Fermentation is a food processing method that extends the shelf life of food, improves quality, and has health benefits (Ji, Zhang, Yuan, et al. 2022; Xu et al. 2024). Current research demonstrates that fermented *Rosa roxburghii* fruit juice exhibits increased total polyphenol content along with enhanced antioxidant capacity and xanthine oxidase inhibitory activity (Zhou, Peng, et al. 2023). Furthermore, it shows potential immunomodulatory effects, intestinal barrier protection, and gut microbiota modulation capabilities (Ji, Zhang, Tang, et al. 2022; Xu et al. 2024). Our preliminary studies have identified multiple bioactive components in RRFJ, including flavonoids and polyphenols, which contribute to its significant anti‐inflammatory and antioxidant properties (Table 1) (Chen et al. 2024). However, the potential hypolipidemic effects of RRFJ remain undetermined. This study therefore provides critical evidence supporting RRFJ's development as a functional food for hyperlipidemia prevention.

**TABLE 1:** Summary of active components in RRFJ.

NO.	Identification name	Molecular formular	No.	Identification name	Molecular formular
1	L‐Threonine	C_4_H_9_NO_3_	23	Procyanidin A1	C_30_H_24_O_12_
2	Gluconic acid	C_6_H_12_O_7_	24	Procyanidin B2	C_30_H_26_O_12_
3	β‐D‐Glucopyranuronic acid	C_6_H_10_O_7_	25	Procyanidin B1	C_30_H_26_O_12_
4	D‐Mannitol	C_6_H_14_O_6_	26	Emodin	C_15_H_10_O_5_
5	Betaine	C_15_H_11_NO_2_	27	4‐Hydroxybenzaldehyde	C_7_H_6_O_2_
6	Trehalose	C_12_H_22_O_11_	28	Catechin	C_15_H_14_O_6_
7	Quinic acid	C_7_H_12_O_6_	29	Homovanillic acid	C_9_H_16_O_4_
8	Trigonelline	C_7_H_7_NO_2_	30	p‐Coumaric acid	C_9_H_8_O_3_
9	L‐Glutamic acid	C_5_H_9_NO_4_	31	Epicatechin	C_15_H_14_O_6_
10	D‐proline	C_5_H_9_NO_2_	32	Chrysin	C_15_H_14_O_7_
11	Arginine	C_6_H_14_N_4_O_2_	33	Protocatechualdehyde	C_7_H_6_O_3_
12	Pantothenic acid	C_9_H_17_NO_5_	34	Cuminaldehyde	C_10_H_12_O
13	L‐Homoserine	C_4_H_9_NO_3_	35	Ellagic acid	C_7_H_6_O
14	Pyrogallol	C_6_H_6_O_3_	36	Ethyl gallate	C_9_H_10_O_5_
15	Ascorbic acid	C_6_H_14_O_6_	37	Taxifolin	C_15_H_12_O_7_
16	Uracil	C_4_H_4_N_2_O	38	Paeonol	C_9_H_10_O_3_
17	Nicotinic acid	C_6_H_5_NO_2_	39	Abscisic acid	C_15_H_20_O_4_
18	Kojic acid	C_6_H_6_O_4_	40	Quercetin	C_15_H_10_O_7_
19	L‐Norleucine	C_6_H_13_NO_2_	41	Morin	C_15_H_20_O_7_
20	Gallic acid	C_7_H_6_O_5_	42	Asiatic acid	C_36_H_48_O_5_
21	Epigallocatechin	C_15_H_14_O_7_	43	Corchorifatty acid F	C_18_H_32_O_5_
22	3‐Indoleacrylic acid	C_11_H_9_NO_2_	44	Phytosphingosine	C_18_H_39_NO_3_

The liver plays a crucial role in energy and substance metabolism, and a diet high in fat can lead to changes in liver metabolite levels and metabolic pathways (Jiang et al. 2022; Zhou, Ma, et al. 2023). Metabolomics involves analyzing metabolites in the body fluids, cells, and tissues of organisms, which can help uncover the underlying causes of many metabolic diseases. It has been shown that the composition of gut flora is closely linked to high‐fat diets (HFDs) like obesity, which can negatively impact the host's metabolism and energy balance (Chen et al. 2018; Johnson et al. 2016; Dubinski et al. 2021). Research has demonstrated that the liver plays a key role in regulating the intestinal microbiota and its effects. Imbalances in the gut flora can directly affect the liver by disrupting hepatic carbohydrate and lipid metabolism through a process known as the enterohepatic axis. This pathway is a major factor in the development and progression of hepatic metabolic disorders, influencing functions such as metabolite production, enterohepatic recycling, and the bacterial end‐products of the gut (Wang, Han, et al. 2022; Yi et al. 2021). This study aims to use metabolomics and microbiota analysis to understand how *Rosa Roxburghii* fermented juice protects against dyslipidemia and lipid deposition through the gut‐liver axis. By providing a deeper theoretical basis for the use of RRFJ as a functional food for preventing hyperlipidemia, this research will shed light on its potential benefits.

## 2 Materials and Methods

### 2.1 Materials Reagents

*Rosa roxburghii* fermented juice (Yin Long) is supplied by Guizhou Shanwangguo Health Industry Co. Ltd. (Guizhou, China). Assay kits for TG, TC, LDL‐C, MDA, and GSH were purchased from Nanjing Jiancheng Biotechnology Research Institute, while the mouse TNF‐α and IL‐6 enzyme‐linked immunosorbent assay (ELISA) kit is from Shenzhen New Biotechnology Co. Ltd. All reagents and chemicals, except for UPLC grade reagents (formic acid, 89 acetonitrile, and methanol from Merck), are of analytical grade.

### 2.2 Animals Experiments and Design

**FIGURE 1:**
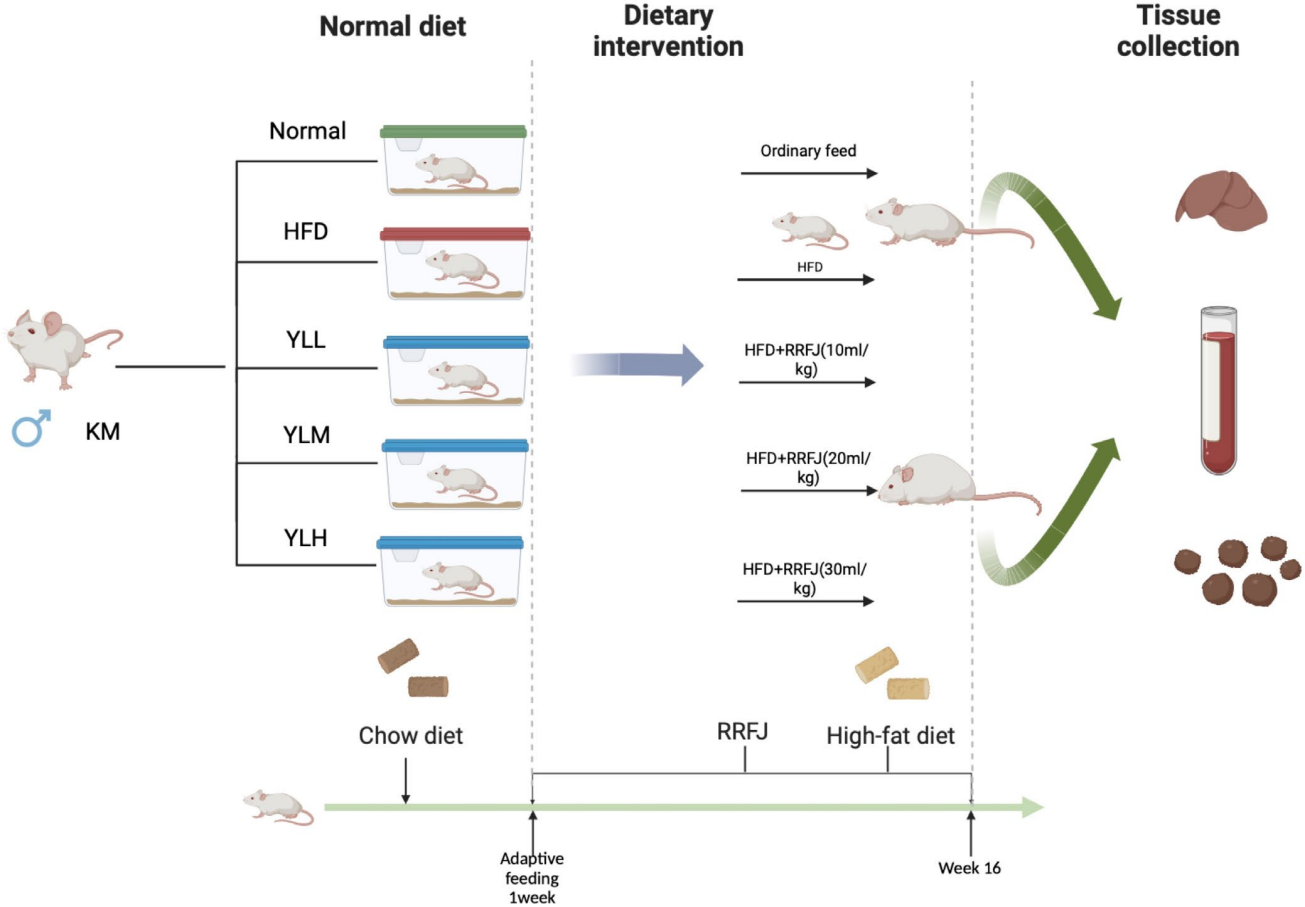
Experimental design diagram.

The study used male KM mice, 6 weeks old, weighing 18–22 g, obtained from the Animal Center of Guizhou Medical University. The mice were kept in standard laboratory conditions with a 12/12 h light/dark cycle, a temperature of 25°C ± 1°C, and a humidity of 60% ± 5%. They had free access to water and food. The animal experiment was conducted in accordance with the Guidelines for the Care and Use of Experimental Animals and was approved by the Ethics Committee of Guizhou Medical University. After one week of acclimatization, 30 mice were randomly divided into 5 groups: a control group (Normal), a model group (HFD), a low‐dose treatment group (YLL), a medium‐dose treatment group (YLM), and a high‐dose treatment group (YLH). The mice in each group were treated as follows: the Normal group received regular feed, the HFD group received a HFD, the YLL group received a HFD and RRFJ by gavage (10 mL/kg), the YLM group received a HFD and RRFJ by gavage (20 mL/kg), and the YLH group received a HFD and RRFJ by gavage (30 mL/kg). The Normal and HFD groups were also given physiological saline as a control. All groups of mice had access to water as usual. The intervention lasted for 16 weeks, with weekly monitoring of mouse weight. The duration of HFD feeding was selected based on prior research demonstrating that a 16‐week HFD reliably induces dyslipidemia in animal models (Nerurkar et al. 2019). Prior to the end of the experiment, the mice fasted overnight, and blood, liver, other organ tissues, and feces were collected for analysis. (The high‐fat feed formula consisted of 67% basic feed, 10% lard, 20% sucrose, 2.5% cholesterol, and 0.5% sodium gallate) (Figure 1).

### 2.3 Organ Index

The collected liver, spleen, kidneys, and brain were washed with physiological saline, dried with filter paper, and accurately weighed. The organ index was calculated using Equation (1):

(1) $$\text{Organ Index}\;\left(100\%\right)=\text{The weight of organ}\;\left(g\right)\times 100/\text{Body weight}\;\left(g\right)$$

### 2.4 Determination of Serum and Liver Biochemical Indicators

The blood sample was allowed to sit at room temperature for 1 h and then centrifuged at 3000 rpm for 10 min to separate the serum portion. A commercial reagent kit from the Nanjing Institute of Biotechnology was utilized to analyze levels of triglycerides (TG), total cholesterol (TC), and low‐density lipoprotein cholesterol (LDL‐C) in both serum and liver, as well as levels of liver malondialdehyde (MAD) and reduced glutathione (GSH). The presence of liver tumor necrosis factor alpha (TNF‐α) and interleukin‐6 (IL‐6) was detected using an enzyme‐linked immunosorbent assay (ELISA) kit.

### 2.5 Liver Histopathology

Liver samples were collected and fixed in 10% paraformaldehyde for 24 h, followed by embedding in paraffin and cutting into 4 μm sections for staining with hematoxylin and eosin (H&E) and oil red O.

### 2.6 Analysis of Gut Microbiota

The genome DNA of the sample was extracted using the CTAB or SDS method, and the purity and concentration of the DNA were confirmed through agarose gel electrophoresis. The DNA was diluted to a concentration of 1 ng/μL with sterile water, and then used as a template for PCR amplification. Special primers with Barcode, Phusion High‐Fidelity PCR Master Mix with GC Buffer from New England Biolabs, and high‐fidelity enzymes were used to ensure efficient and accurate amplification. The bacterial 16 s rRNA gene V3‐V4 region was amplified using forward primer 515F (5’‐GTGCCAGCMGCCGCGGTAA‐3′) and reverse primer 806R (5’‐GGACTACHVGGGTWTCTAAT‐3′). The PCR products were analyzed on a 2% agarose gel, purified, quantified, mixed in equal amounts, and then subjected to another round of 2% agarose gel electrophoresis for further analysis. The rubber recovery kit provided by Qiagen was used to recycle the target strip, and the TruSeq DNA PCR‐Free Sample Preparation Kit was utilized for library construction. The library was quantified using Qubit and Q‐PCR, and then sequenced on the NovaSeq6000 platform.

### 2.7 Liver Metabolomics Analysis

A 100 mg liver sample was extracted using 0.5 mL of a pre‐cooled aqueous methanol/acetonitrile solution (2:2:1, v/v/v), homogenized, and vortex sonicated for 15 min at a low temperature. To remove proteins, the mixtures were stored overnight at −20°C, then centrifuged for 15 min at 15000 rpm, and the supernatants were collected. The supernatants from fecal samples were filtered through a 0.22 μm membrane and injected into a sample vial for metabolomics analysis. Similarly, metabolomics analysis was conducted using UHPLC‐HESI‐Q‐Exactive Plus Orbitrap‐MS with a ZORBAX Eclipse Plus C18 column (2.1 × 100 mm^2^, 1.8 μm; Agilent Technologies, USA). The optimized gradient included H_2_O (0.1% formic acid, A) and acetonitrile (0.1% formic acid, B) with the following specifications: 0–2.5 min, 2%–2% B; 2.5–5 min, 2%–40% B; 5–12 min, 40%–100% B; 12–16 min, 100%–100% B; 16–16.1 min, 100%–2% B; 16.1–19 min, 2%–2% B. The flow rate was 0.3 mL/min. The ion spray voltage was set at 3.5/2.8 kV (+/−), and the scan range was 100–1500 m/z. The auxiliary gas heater and capillary temperatures were set at 350°C and 320°C, respectively. Dynamic exclusion was set for 3 s, and the S‐lens RF level was 50. Data pretreatments, such as peak recognition, peak matching, and retention time correction, were performed using Compound Discoverer 3.2 software. OPLS‐DA and PCA were conducted using SIMCA‐P 14.1 software. Endogenous metabolites were identified by comparing primary and secondary mass spectral fragmentation information using KEGG and HMDB databases, as well as an in‐house metabolite fragment spectrum library for verification through MS2 spectra. MetaboAnalyst 5.0 was utilized to analyze metabolism related to hyperlipidemia after RRFJ preventive therapy.

### 2.8 Statistical Analysis

Data analysis was performed using IBM SPSS Statistics 27 and GraphPad Prism 8.0 software, and results were expressed as mean ± standard deviation. Statistical significance was considered at *p* < 0.05, with *p* < 0.01 indicating very significant results.

## 3 Results

### 3.1 Effects of RRFJ on Body Weight and Blood Lipids in Serum of HFD Mice

**FIGURE 2:**
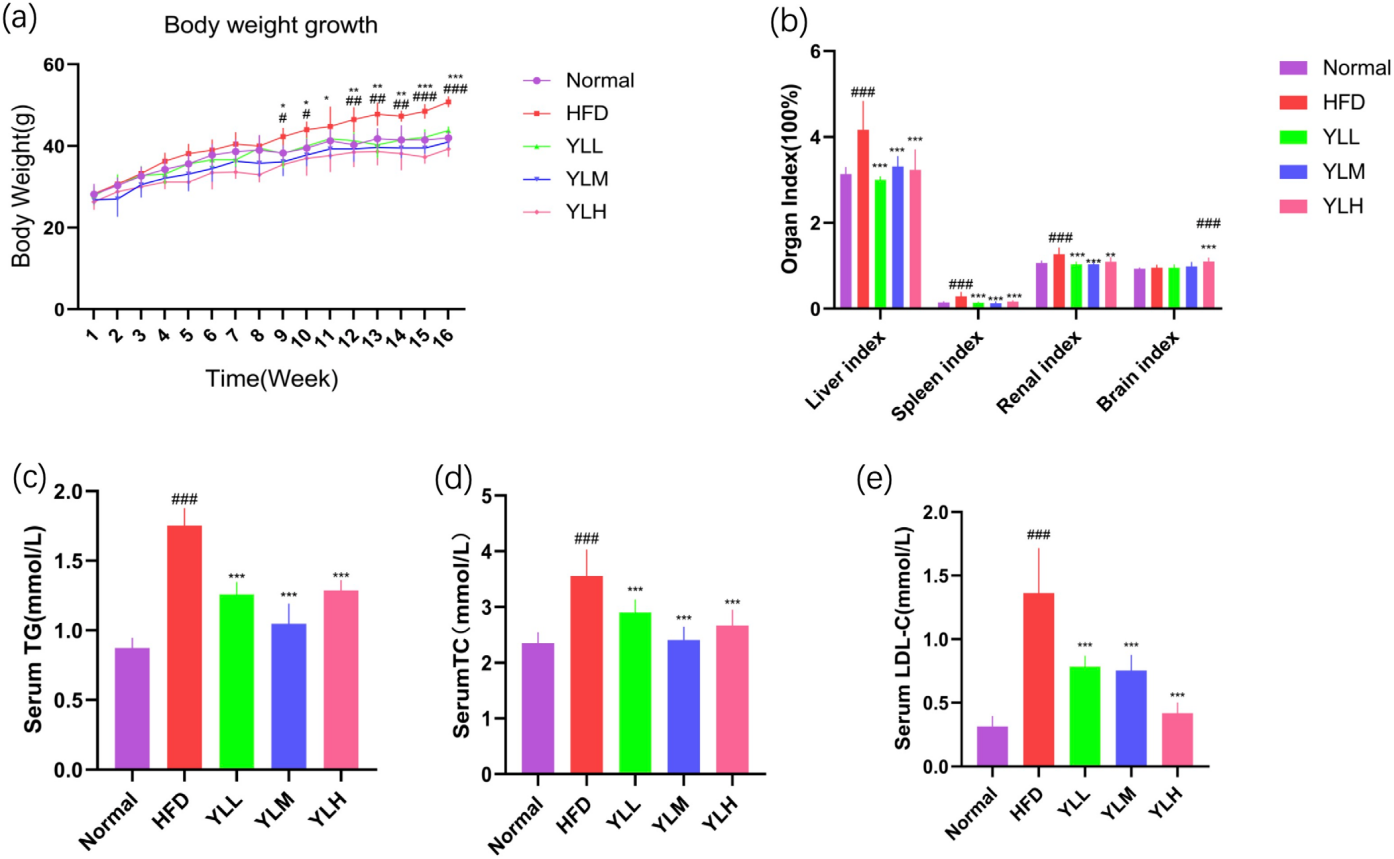
Changes in weekly body weight of mice during the feeding period (a). Mouse organ index (b). Serum TG (c) levels. Serum TC (d) levels. Serum LDL‐C (e) levels. *p < 0.05, **p < 0.01, ***p < 0.001 versus the HFD group. #*p* < 0.05, ##*p* < 0.01, ###*p* < 0.001 versus the Normal group.

Figure 2a shows that there was no significant difference in weight gain among the groups initially. However, after 9 weeks of feeding, mice on a HFD had a significantly higher body weight compared to other groups. Oral administration of different doses of RRFJ effectively prevented weight gain induced by the HFD, with YLH showing the most significant inhibitory effect on weight gain. The liver index, spleen index, and kidney index of mice in the HFD group were significantly higher compared to those in the Normal group, indicating fat accumulation in mouse tissues due to the HFD. However, after administering different doses of RRFJ, all indices were significantly lower than those in the HFD group, suggesting that RRFJ can reduce tissue fat accumulation caused by the HFD (Figure 2b). This confirms the successful establishment of the model. The impact of RRFJ on blood lipid levels in mice is depicted in Figure 2c–e. Serum total cholesterol (TC), triglycerides (TG), and low‐density lipoprotein (LDL) levels in the HFD group were significantly higher than those in the control group. The RRFJ group effectively mitigated the elevation of blood lipids induced by the HFD, indicating that RRFJ can improve blood lipid abnormalities caused by a HFD.

### 3.2 The Effect of RRFJ on Liver Lipid Metabolism, Oxidative Damage, and Inflammatory Factors in HFD Mice

**FIGURE 3:**
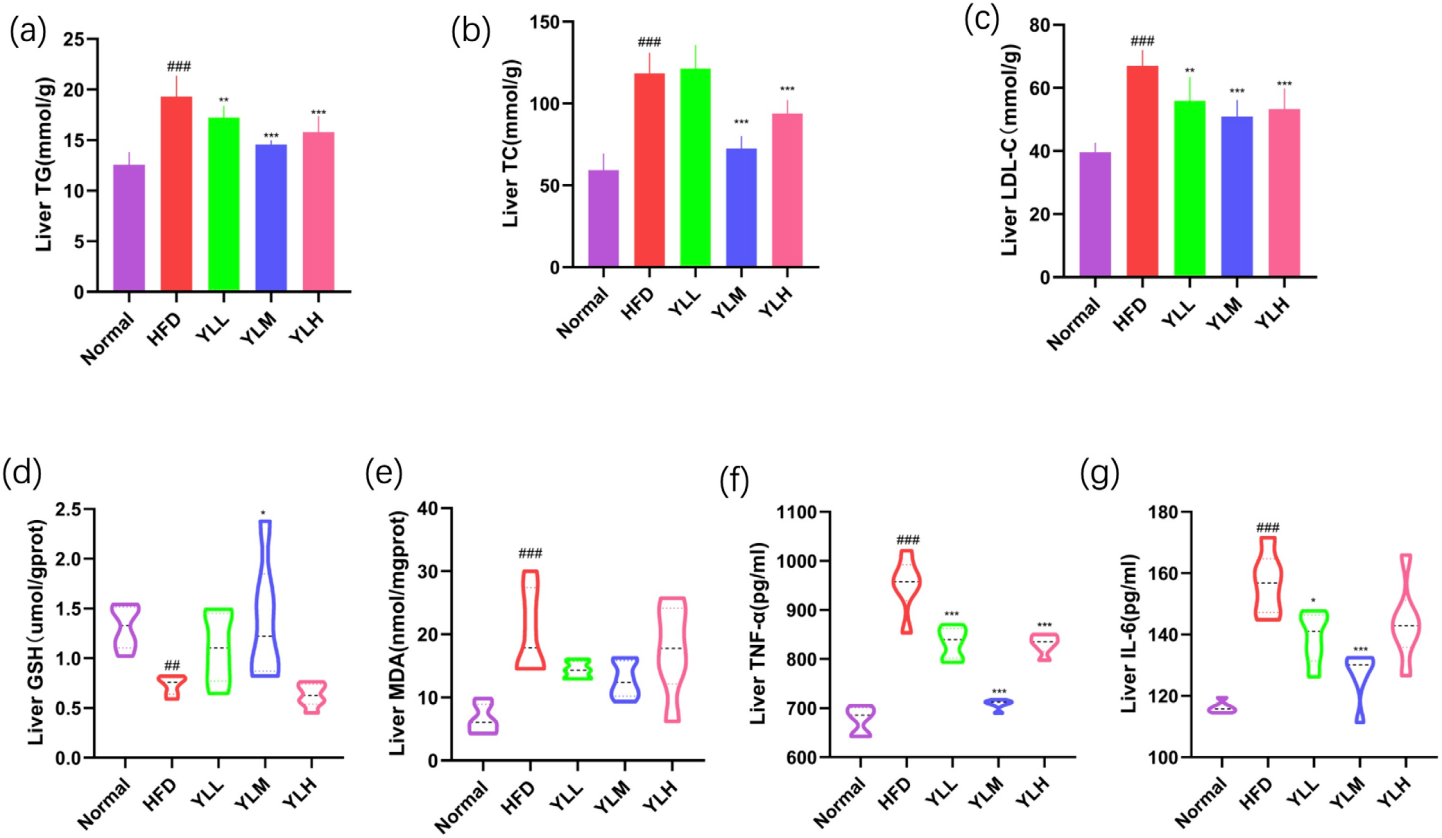
Liver lipid metabolism level: TG (a), TC (b), LDL‐C (c). Liver oxidative stress levels: GSH (d), MDA (e). Liver inflammatory factor levels: TNF‐α (f), IL‐6 (g). Note: *N* = 6, mean ± SEM. *p < 0.05, **p < 0.01, ***p < 0.001 versus the HFD group. ##*p* < 0.01, ### *p* < 0.001 versus the normal group.

To further explore the effects of RRFJ on the liver of mice fed a HFD, lipid metabolism, oxidative stress, and inflammatory factors in the liver were measured. As shown in Figure 3a–c, levels of TG, TG, and LDL‐C in the liver of hyperlipidemic mice induced by a HFD were significantly higher than those in the Normal group. Intervention with RRFJ significantly reduced the levels of these three factors, indicating that RRFJ intervention can alleviate lipid accumulation caused by a HFD. In Figure 3d,e, it is observed that high‐fat feeding decreased the activity of antioxidant glutathione (GSH) and increased the content of the final product of lipid oxidation, malondialdehyde (MDA), suggesting impaired liver oxidative stress. RRFJ intervention can alleviate the decrease in liver GSH caused by the HFD and significantly reduce the increase in liver MDA levels caused by the HFD, thereby alleviating oxidative stress damage. In terms of inflammation markers, there was no significant change in TNF‐α levels in the HFD group compared to the normal group. However, intervention with RRFJ led to a reduction in TNF‐α levels (Figure 3f). Additionally, YLM intervention significantly decreased liver IL‐6 levels compared to the HFD group (Figure 3g).

### 3.3 Effect of RRFJ on Liver Histopathology in HFD Mice

**FIGURE 4:**
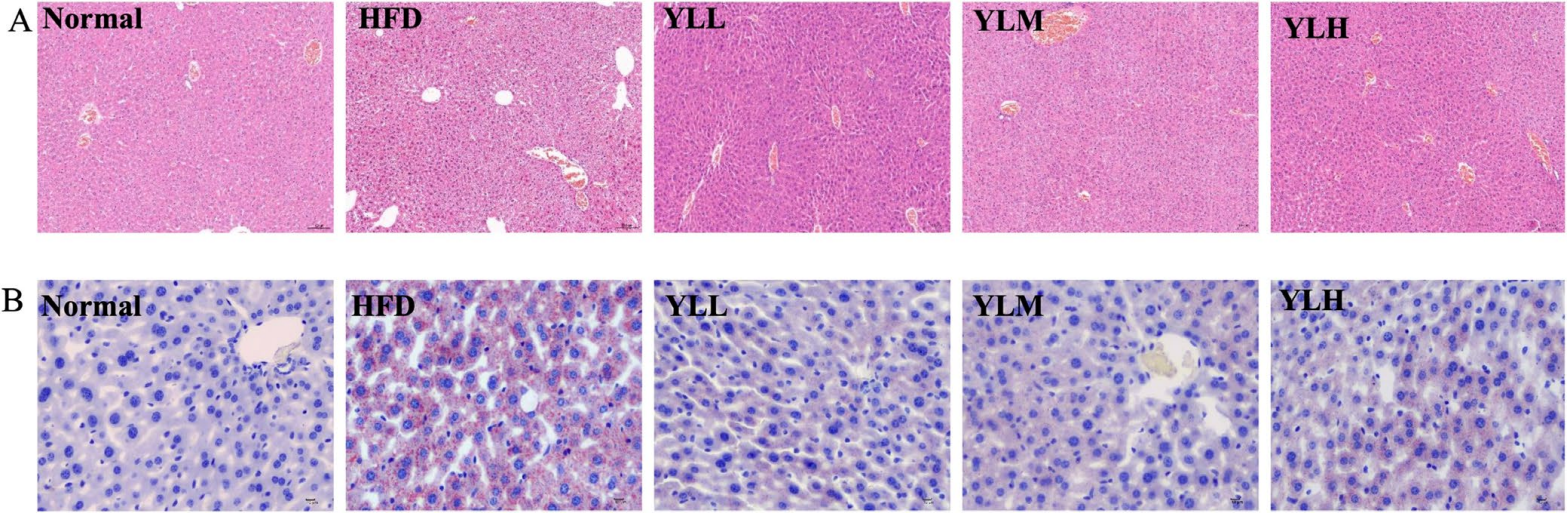
(A) Liver section H&E staining (100×). (B) Liver slices stained with oil red O (400×).

The normal group exhibited intact liver tissue structure with neatly arranged liver cells of uniform size and no fat degeneration. In contrast, the liver cells in the HFD group mice were disordered, showing severe steatosis and a large number of fat vacuoles. However, the liver tissue structure of the mice treated with RRFJ intervention remained intact, with a significant reduction in fat vacuoles (Figure 4A). Oil Red O staining revealed that the Normal group had a small amount of lipid droplets in the liver slices, while the HFD group had a high accumulation of lipid droplets (Figure 4B). The RRFJ group effectively reduced the number and size of liver lipid droplets, with the YLM group showing the most significant effect. These results indicate that RRFJ successfully improves liver lipid deposition induced by a HFD.

### 3.4 RRFJ Can Reverse the Imbalance of Gut Microbiota in HFD Mice

**FIGURE 5:**
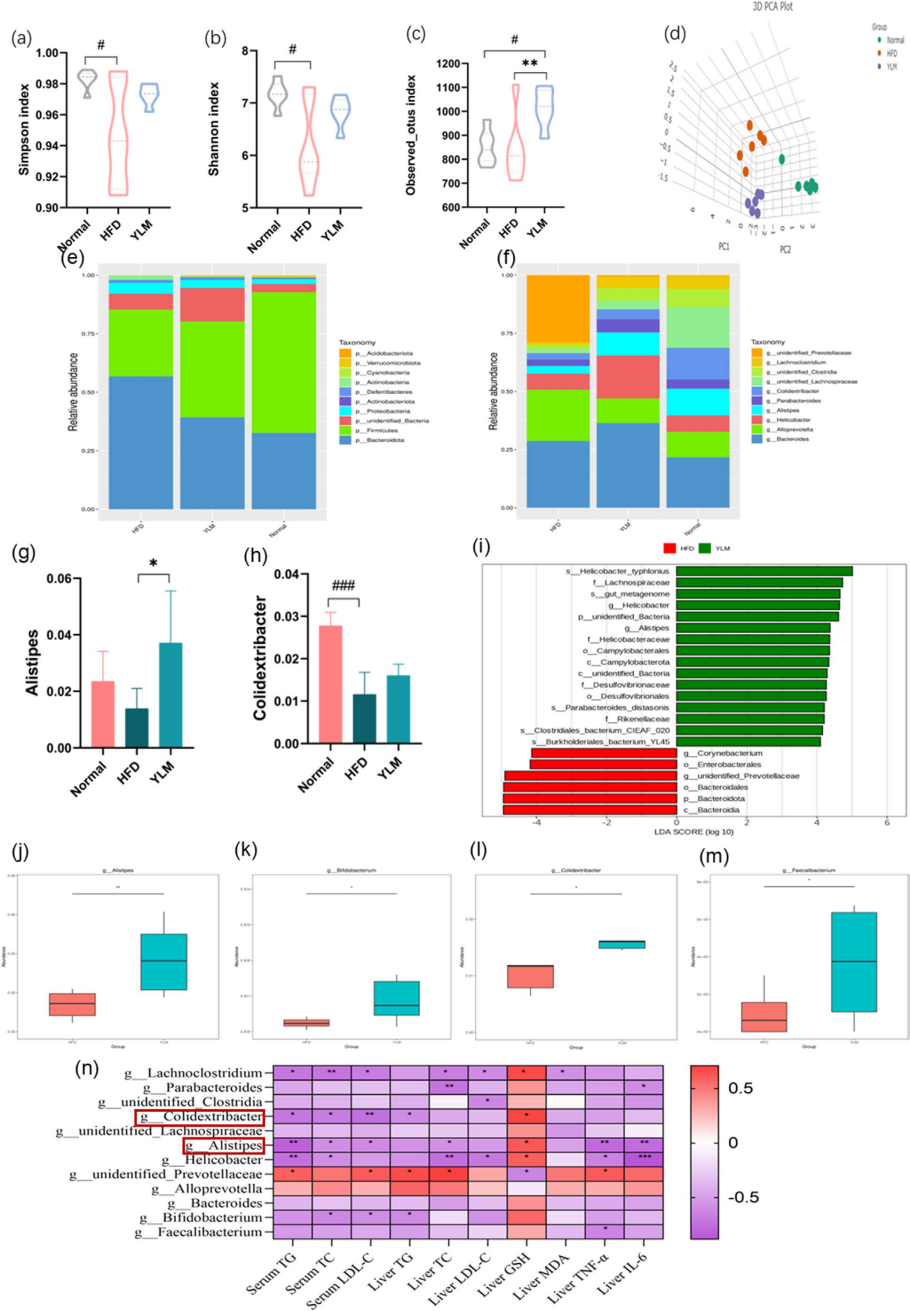
Composition of gut microbiota. (a) The alpha diversity of simplicity index. (b) The alpha diversity of Shannon index. (c) The alpha diversity of observed otus index. (d) The relative abundance of 3D PCA. (e) The relative abundance at the phylum level. (f) Relative abundance at the genus level of microorganisms. (g) The abundance of *Alistipes* genus. (h) The abundance of *Colidextribacter* genus. (i) LDA value distribution bar chart based on genus level. (j–m) Statistical chart of significant differences in species Metastats between groups based on genus level: *Alistipes* genus (j), *Bifidobacterium* genus (k), *Colidextribacter* genus (l), *Faecalibacterium* genus (m). (n) Using Spearman correlation analysis, the correlation between 12 gut microbiota and 10 physiological parameters in HFD versus YLM, with red representing upregulation and purple representing downregulation. *p < 0.05, **p < 0.01, ***p < 0.001. #*p* < 0.05, ##*p* < 0.01, ###*p* < 0.001.

The intestinal microbiota plays a crucial role in maintaining intestinal health and balance. In order to study the impact of FFRJ on the gut microbiota of mice fed a HFD, we conducted high‐throughput 16S rDNA sequencing on the fecal samples of mice in the Normal, HFD, and YLM groups. We initially compared the microbial diversity among the groups using alpha diversity metrics such as the Simpson index (Figure 5a), Shannon index (Figure 5b), and observed otus index (Figure 5c). The Kruskal‐Wallis test showed significantly lower Simpson and Shannon indices in the HFD group versus the Normal group, with recovery after RRFJ treatment. Observed OTUs decreased in HFD but increased in YLM, suggesting RRFJ's role in restoring microbial richness and diversity in HFD‐fed mice. Beta diversity analysis, which compares the composition of microbial communities between samples, revealed distinct microbial community compositions among the groups as depicted in the PCA plot (Figure 5d). This suggests that YLM has the potential to modulate the gut microbiota of HFD mice.

The top ten gates with abundance rankings at the phylum level are as follows: *Bacteroidota*, *Firmicutes*, *unidentified‐Batteria*, *Proteobacteria*, *Actinobacteriota*, *Deferreribacters*, *Actinobacteria*, *Cyanobacter*, *Verrucomimicrobiota*, and *Acidobacteriota* (Figure 5e). The abundance of *Bacteroidota* was significantly increased in the HFD group compared to the Normal group, while the abundance of *Firmicutes* was significantly reduced. This imbalance was corrected after YLM intervention. At the genus level (Figure 5f), the fecal microbiota of mice mainly consists of *Bacteroides*, *Alloprevotella*, *Helicobacter*, *Alistipes*, *Parabcteroides*, *Colidextribacter*, *unidentified_Lachnospiraceae*, *unidentified_Clostridia*, *Lachnoclostridium*, and *unidentified_Prevotellaceae*. *Bacteroides* and *Allophorotella* were upregulated in the HFD group compared to the Normal group, while *Helicobacter*, *Alistipes*, *Parabcteroides*, and *Colidextribacter* were downregulated. Following YLM intervention, *Parabcteroides*, *Alistipes* (Figure 5g), and *Colidextribacter* (Figure 5h) were restored. Further analysis using LEfSe and MetaStates with LDA scores > 3 and *p* < 0.05 identified differential biomarkers. The LEfSe analysis (Figure 5i) showed the differential taxa between the groups. The results showed that YLM intervention significantly increased the relative abundance of *Alistipes*, *Bifidobacterium*, *Colidextribacter*, and *Faecalibacterium* genera in the feces of HFD‐induced mice at the genus level (Figure 5j–m).

The researchers used Spearman correlation analysis to examine the relationship between biochemical markers in serum and liver and the major microbial communities. They found that the *Alistipes* genus had a negative correlation with serum biochemical markers and liver inflammatory factors, but a positive correlation with liver GSH. The *Parabacteroides* genus was negatively correlated with liver TG and liver IL‐6. The *Colidextribacter* genus showed a negative correlation with serum lipid markers. The Bifidobacterium genus had a negative correlation with serum TC, serum LDL‐C, and liver TG. On the other hand, the *Faecalibacterium* genus had a positive correlation with liver TNF‐α. The study concluded that *Alistipes* and other gut microbiota are closely linked to biochemical markers in serum and liver (Figure 5n).

### 3.5 Effect of RRFJ on Liver Metabolomics in Mice

**FIGURE 6:**
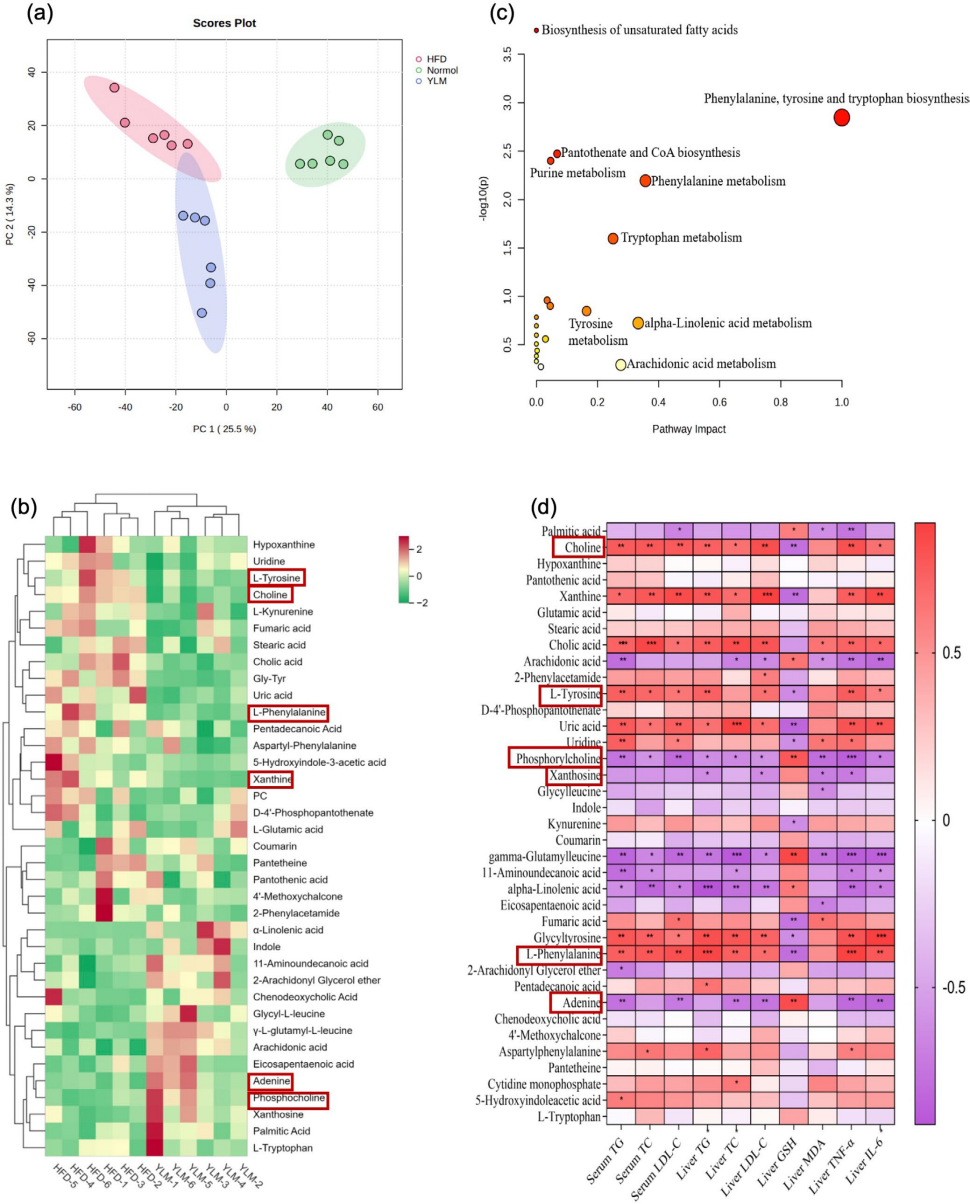
Liver metabolome. PCA diagram of normal versus HFD versus YLM (a). Heat map of differentially expressed metabolites in liver metabolites (b). Pathway enrichment of differentially expressed metabolites in liver and liver metabolites (c). Heat map (d) the correlation analysis between differentially expressed metabolites in liver metabolites and physiological indicators such as blood lipids, with red representing upregulation and purple representing downregulation *p < 0.05, **p < 0.01, ***p < 0.001.

The lipid‐lowering effect of RRFJ on mouse serum and liver, as well as its impact on liver histopathology, led to the selection of liver samples from Normal, HFD, and YLM groups of mice for liver metabolomics analysis. The PCA score plot in Figure 6a shows the changes in liver metabolism profiles between the Normal, HFD, and YLM groups. The separation between the Normal and HFD groups indicates alterations in endogenous metabolites in the liver of mice due to lipid deposition, while YLM intervention was found to modify liver metabolism induced by a high‐fat diet in HFD mice. By analyzing metabolites with VIP > 1.5, *p* < 0.05, and FC > 1.2 or < 0.833, 37 differential metabolites were identified in the YLM and HFD groups, with 15 upregulated and 22 downregulated metabolites after YLM intervention (Figure 6b). These findings suggest that RRFJ can improve the liver metabolism profile of mice with metabolic dyslipidemia. Furthermore, MetaboAnalyst 5.0 was used for metabolic pathway enrichment analysis, revealing key metabolic pathways such as biosynthesis of phenylalanine, tyrosine, and tryptophan, phenylalanine metabolism, tryptophan metabolism, alpha‐linolenic acid metabolism, arachidonic acid metabolism, tyrosine metabolism, pantothenate and CoA biosynthesis, purine metabolism, and biosynthesis of unsaturated fatty acids (Figure 6c).

The Spearman correlation analysis was used to identify 37 differential metabolites and 10 metabolic parameters for further correlation analysis. The aim was to investigate the relationship between the regulatory effects of YLM on metabolites and its beneficial effects on regulating blood lipid abnormalities. The results (shown in Figure 6d) revealed that 24 metabolites were significantly correlated with at least one metabolic parameter level. Among these, metabolites such as choline, xanthine, cholic acid, L‐tyrosine, uric acid, uridine, and L‐phenylalanine were found to be associated with serum lipid levels, liver lipid levels, as well as liver MDA and TNF‐α levels. The level of IL‐6 showed significant positive and negative correlations with the level of liver GSH. Additionally, metabolites like phosphorylcholine, γ‐L‐glutamyl‐L‐leucine, α‐linolenic acid, and adenine were linked to blood lipid levels in serum and liver, as well as liver MDA and TNF‐α levels. The level of IL‐6 exhibited significant negative and positive correlations with the level of liver GSH. Amino acids and other compounds were identified as crucial for YLM in preventing lipid deposition caused by a HFD.

### 3.6 Correlation Analysis of Liver Differential Metabolites and Differential Bacterial Communities

**FIGURE 7:**
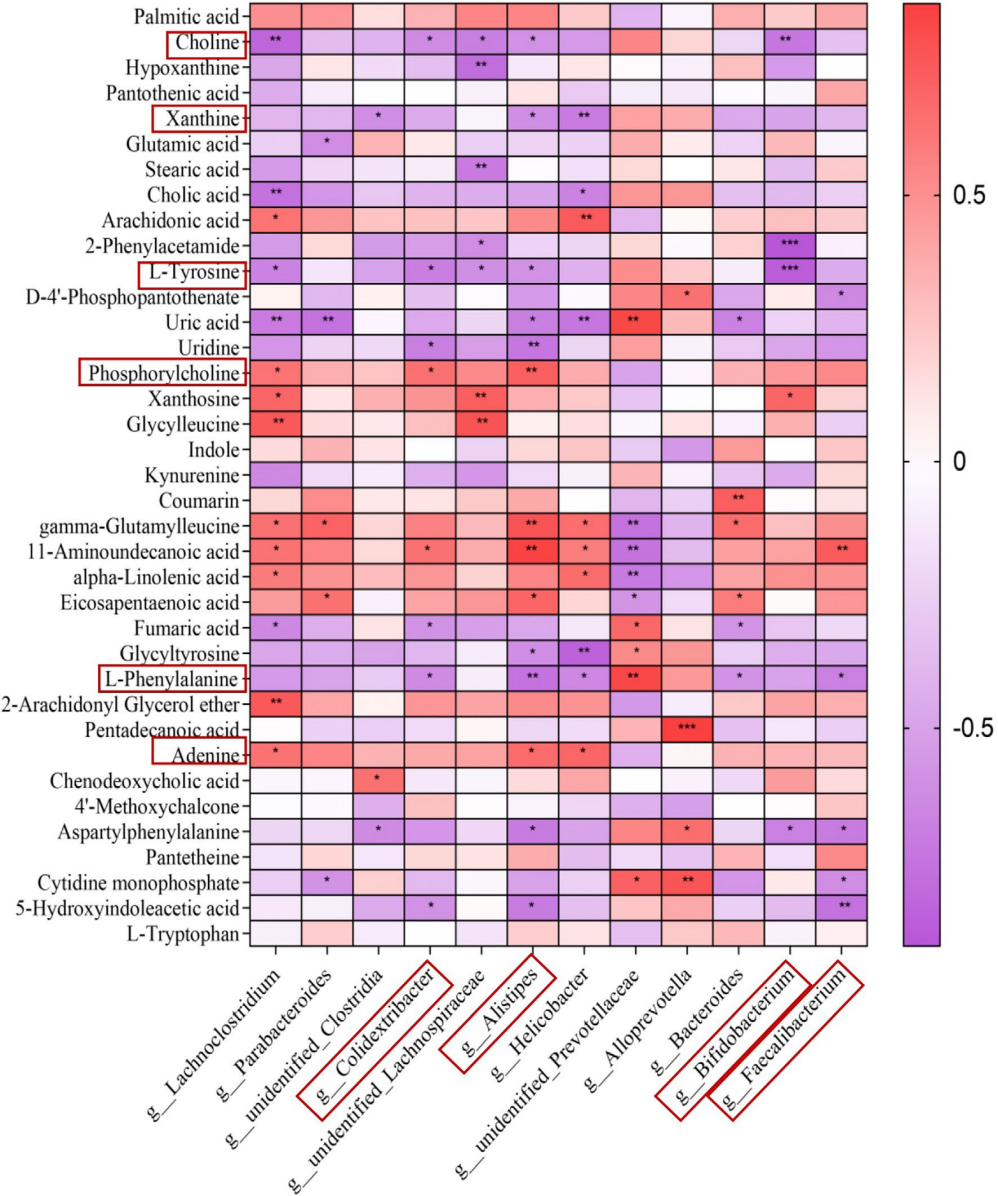
Using Spearman correlation analysis, 37 liver differential metabolites and 12 gut microbiotas were correlated in HFD versus YLM, with red representing upregulation and purple representing downregulation. *p < 0.05, **p < 0.01, ***p < 0.001.

To further elucidate the relationship between gut microbiota and metabolites in the liver following RRFJ intervention, a correlation analysis was performed on 37 different metabolites and 12 gut microbiota. The results showed a significant correlation between 30 metabolites and at least one gut microbiota (Figure 7). Genera such as *Colidextribacter*, *Alistipes*, and *Bifidobacterium* were found to be negatively correlated with metabolites like Choline, L‐Tyrosine, Uric acid, and L‐Phenylalanine, while being positively correlated with metabolites such as Phosphocholine and 11‐Aminoundecanoic acid. On the other hand, the *Faecalibacterium* genus showed a negative correlation with D‐4′‐Phosphopantothenate, Uric acid, Aspartyl Phenylalanine, and PC, but a positive correlation with 11 Aminoundecanoic acid. These findings suggest that RRFJ may help prevent dyslipidemia in the serum and liver by modulating the levels of *Colidextribacter*, *Alistipes*, *Bifidobacterium*, and *Faecalibacterium* genera.

## 4 Discussion

The incidence of hyperlipidemia is increasing due to changes in diet and lifestyle. HFDs are a major risk factor for obesity, diabetes, fatty liver, atherosclerosis, and other metabolic diseases (Wang, Kong, et al. 2020). Long‐term consumption of HFDs can lead to obesity and hyperlipidemia by causing fat accumulation in the body (Wen et al. 2023). In this study, our 16‐week HFD model effectively recapitulated key features of human dyslipidemia, including elevated TG, TC, and LDL‐C in serum (Nerurkar et al. 2019; Shen et al. n.d.). It was found that RRTJ can effectively prevent lipid abnormalities caused by HFDs in mice. RRFJ intervention for 16 weeks prevented weight gain, liver enlargement, and elevated levels of cholesterol and triglycerides in the blood and liver of mice on a HFD. This is consistent with previous research (Xu et al. 2023; Li, Huang, et al. 2021). HFDs can increase oxidative stress in the liver, leading to liver injury and fibrosis. RRFJ was found to reduce oxidative stress and inflammation in the liver of mice on a HFD, preventing liver damage. Histopathological observations showed significant fat accumulation in the livers of mice on a HFD, with RRFJ treatment reducing liver fat. The YLM treatment group showed the best preventive effect among the treatment groups. In our study, we observed that administering different doses of RRFJ to HFD mice did not show a dose‐dependent effect on blood lipid indicators. However, YLM exhibited the best preventive effect, suggesting that RRFJ can effectively prevent serum and liver lipid abnormalities induced by a HFD. It also helps in inhibiting liver oxidative stress, reducing levels of inflammatory factors in HFD mice, and alleviating liver lipid deposition.

The gut microbiota represents the largest symbiotic ecosystem in the human body, playing a crucial role in host metabolism of both substances and energy. Research has demonstrated that in addition to factors such as obesity, genetic predisposition, and pancreatic islet dysfunction, excessive nutritional intake can significantly alter the composition of gut microorganisms, leading to the predominance of pathogenic microbial species (Ma et al. 2019). Recent studies have highlighted the importance of regulating gut microbiota to treat dyslipidemia, NAFLD, and other obesity‐related diseases (Huang 2020; Shi et al. 2023). Our analysis revealed significant changes in the gut microbiota structure in HFD mice compared to the Normal and NFD groups. YLM intervention significantly improved the disruption of gut microbiota structure caused by HFD. Specifically, we observed an increase in the abundance of *Bacteroides* in the HFD group, which is associated with a diet high in animal fat and protein (Hayouka and Tirosh 2020). YLM intervention reversed the lower abundance of *Alistipes*, *Parabacteroides*, and *Colidextribacter* in HFD mice. *Alistipes*, in particular, plays a crucial role in preventing obesity and is positively correlated with SCFA levels (Li, Wang, et al. 2022). *Alistipes* produces acetate and mediates lipid metabolism in HFD mice via the gut microbiota‐acetate axis, thereby ameliorating dyslipidemia (Yin et al. 2018). Additionally, the low abundance of *Parabacteroides* and *Colidextribacter* genera in the HFD group aligns with previous research findings (Du et al. 2022). The MetaStat analysis results indicated that the YLM intervention significantly increased the levels of *Bifidobacterium* and *Faecalibacterium* genera in the feces of mice fed a HFD. Previous studies have shown that HFDs can disrupt the balance between these two genera, with *Bifidobacterium* levels decreasing, which is a beneficial bacterium associated with lower obesity risk (Wang, Kong, et al. 2020; Xiao et al. 2022). Following the administration of RRFJ, there was a notable increase in the abundance of microbial communities such as *Alistipes*, *Parabacteroides*, *Colidextribacter*, *Bifidobacterium*, and *Faecalibacterium*.

To systematically investigate correlations between the identified bacterial genera and host metabolic outcomes. Spearman correlation analysis revealed that these parameters were significantly associated with the improvement of hyperlipidemia and related metabolic indices. The study found that *Alistipes* reduces inflammation and shows a negative correlation with blood lipid markers such as TC and TG, which is consistent with our findings (Bi et al. 2023; Li 2021). Bile acids are synthesized in the liver and play a key role in regulating lipid absorption and metabolism. Studies have demonstrated that *Parabacteroides* exhibits a robust capacity to convert primary bile acids into secondary bile acids, through modulation of bile acid metabolism, *Parabacteroides* exerts multiple beneficial effects, including regulation of lipid metabolism and inflammation, notably, it shows negative correlations with fat accumulation and key metabolic parameters such as TC, TG, and IL‐6 levels (Hu et al. 2022; Zhu et al. 2022; Wang et al. 2019). Previous studies reported that *Colidextribacter* was significantly enriched in HFD‐induced hyperlipidemic mice, showing a significant correlation with serum MDA levels, a well‐established biomarker of lipid peroxidation, notably, reversal of *Colidextribacter* abundance effectively reduced lipid accumulation and ameliorated intestinal barrier impairment (Wang et al. 2021; Yan et al. 2022). In the present study, *Faecalibacterium* demonstrated negative correlations with blood lipid levels and inflammatory responses, which aligns with previous findings (Zhu et al. 2020). As a quintessential butyrate‐producing bacterium within the gut microbiota, *Faecalibacterium*‐derived butyrate exerts multifaceted protective effects through: remodeling gut microbial composition and enhancing intestinal barrier integrity; ameliorating fatty acid peroxidation; and activating the AMPK‐ACC signaling pathway, thereby alleviating hepatic lipid accumulation and oxidative stress (Zhu et al. 2020; Xue et al. 2021; Khan and Jena 2015). Collectively, these results demonstrate that RRFJ administration rectifies HFD‐driven gut microbiota dysbiosis, leading to significant improvements in lipid metabolism, hepatic oxidative damage, and inflammation, ultimately retarding metabolic disorder progression. The consistent alterations in *Alistipes*, *Parabacteroides*, *Faecalibacterium*, and *Colidextribacter* abundances position these taxa as promising candidate biomarkers for metabolic diseases including dyslipidemia, T2DM, and obesity.

The gut microbiota dynamically modulates hepatic metabolomic profiles through the enterohepatic circulation of microbial‐derived metabolites, thereby bridging microbial ecology with liver metabolic homeostasis. Lipid metabolism disorder refers to an abnormal blood lipid profile and liver lipid distribution, with the liver playing a crucial role in body metabolism (Li, Wang, et al. 2022). Using omics methods, the regulatory effect of RRFJ on liver metabolism in HFD mice was explored. A total of 38 differential metabolites were identified in mouse liver metabolomics analysis. Downregulated metabolites included L‐Tyrosine, Choline, Stearic acid, L‐Kynurenine, L‐Phenylalanine, Xanthine, Gly‐Tyr, and others. Up‐regulated metabolites included Arachidonic acid, Adenine, Phosphocholine, and γ‐L‐glutamyl‐L‐leucine, among others. Research has shown that phenylalanine hydroxylase converts L‐phenylalanine into L‐tyrosine, which has been identified as a potential biomarker for hyperlipidemia. In a state of hyperlipidemia, the content of L‐tyrosine increases, leading to oxidative stress and DNA damage (De Prá et al. 2014; Fernstrom and Fernstrom 2007; Shang et al. 2024). L‐Tyrosine and L‐Phenylalanine are central metabolites in the biosynthesis of Phenylalanine, Tyrosine, and Tryptophan metabolic pathways, as well as Phenylalanine metabolism pathways (Yanan et al. 2021). RRFJ intervention in this study alleviated the levels of L‐Tyrosine and L‐Phenylalanine in the liver of HFD mice. Additionally, a deficiency in Choline can induce NASH (Matsumoto et al. 2013), while a HFD can lead to a significant increase in the levels of hypoxanthine and xanthine in the body (Wei et al. 2021). Xanthine and Adenine are both central metabolites in the purine metabolism pathway, and their metabolism was found to be abnormal in HFD mice, consistent with previous findings that hyperlipidemia patients often have purine metabolism disorders (Wen et al. 2023). Arachidonic acid is often thought to be a pro‐inflammatory factor that contributes to inflammation in HFD intake. However, animals treated with ARSCO showed an increase in plasma LXA4 levels, indicating that Arachidonic acid actually has significant anti‐inflammatory effects (Gundala and Das 2019). Surprisingly, after administering YLM in this study, the levels of Arachidonic acid in the liver metabolism of HFD mice increased, suggesting that YLM may reverse the low expression of Arachidonic acid in the liver of HFD mice and exert anti‐inflammatory effects. Previous studies have shown a strong correlation between Phosphorylcholine and neuroinflammatory genes (Li et al. 2023), and this study found that YLM reversed Phosphorylcholine in the liver of HFD mice. In addition to participating in various metabolic pathways, the metabolites improved by YLM also play a role in tryptophan metabolism, alpha‐linolenic acid metabolism, arachidonic acid metabolism, tyrosine metabolism, pantothenate and CoA biosynthesis, and biosynthesis of unsaturated fatty acids. RRFJ could potentially be a new type of functional food for preventing lipid abnormalities caused by a HFD.

By exploring relevant metabolites of lipid abnormalities in the liver, correlation analysis using the Spearman method confirmed a significant correlation between liver metabolism and physiological parameters of lipid abnormalities. RRFJ may help prevent dyslipidemia by affecting metabolites such as Xanthine, Cholic acid, L‐Tyrosine, Uric acid, Uridine, and L‐Phenylalanine, which have been found to be closely related to physiological indicators such as TC, TG, and LDL‐C in serum and liver, playing a crucial role in lowering blood lipids. Cholic acid, a primary bile acid present in the liver, is metabolized in the intestine via both the classical pathway mediated by CYP7A1 and the alternative pathway involving CYP27A1; it subsequently activates metabolic receptors such as FXR and TGR5 in the intestine and liver, thereby regulating host metabolism in hyperlipidemia induced by HFD through the enterohepatic circulation (Li, Cui, et al. 2021; Wang et al. 2023). Hepatic bile acid accumulation leads to suppressed FXR expression during inflammation. FXR exerts anti‐inflammatory effects by inhibiting NF‐κB and AP‐1 through multiple signaling pathways (Bertolini et al. 2022). Consistent with the strong positive correlation between bile acids and liver inflammation in this study, RRFJ likely suppresses hepatic inflammation in HFD mice via microbiota‐driven bile acid regulation and FXR activation. Aromatic amino acids (AAAs) critically influence systemic metabolism and liver function. Increased L‐Tyrosine indicates mitochondrial dysfunction, whereas elevated L‐Phenylalanine compromises insulin sensitivity, collectively contributing to diet‐induced metabolic inflammation (Xie et al. 2023). In this study, L‐Tyrosine and L‐Phenylalanine were highly expressed in the HFD group and showed significant positive correlations with TC, TG, TNF‐α, and IL‐6. RRFJ intervention suppressed their levels, further suggesting that RRFJ may reduce hepatic lipid accumulation and inflammation in HFD‐fed mice by improving the liver metabolic profile. Further analysis and research are necessary to fully understand the mechanisms at play.

Endotoxins and other bacterial byproducts from the intestines travel to the liver through the gut‐liver axis and can stimulate harmful immune responses and inflammation, which can worsen obesity. To explore the connection between gut bacteria and liver metabolites after RRFJ intervention, a correlation analysis was conducted on the different metabolites in the liver and the main gut bacteria. The results showed a significant correlation between 30 metabolites and at least one type of gut bacteria. The *Alistipes* genus was found to have a negative correlation with choline, xanthine, L‐tyrosine, L‐phenylalanine, and a positive correlation with phosphocholine and adenine. The *Colidextribacter* genus showed a similar negative correlation with choline, L‐tyrosine, L‐phenylalanine, and a positive correlation with phosphocholine. *Bifidobacterium* was negatively correlated with L‐tyrosine, while *Faecalibacterium* was negatively correlated with L‐phenylalanine. From a biochemical perspective, choline is first phosphorylated to form phosphocholine, which subsequently undergoes conversion to phosphatidylcholine (PC) via the CDP‐choline pathway; a deficiency in PC biosynthesis impairs hepatic very‐low‐density lipoprotein (VLDL) assembly and secretion, consequently leading to compromised TG export from the liver (Roy et al. 2022; Song et al. 2025). The gut microbiota metabolizes choline and L‐carnitine to produce trimethylamine (TMA), most of which is absorbed into the bloodstream and rapidly oxidized by hepatic enzymes into trimethylamine N‐oxide (TMAO); both TMA and TMAO can contribute to the development of fatty liver disease (Romano et al. 2017; Chu et al. 2019). Dysbiosis of the gut microbiota may contribute to hepatic choline excess (Chen, Liu, et al. 2016). *Alistipes*, *Colidextribacter*, and *Bifidobacterium* demonstrate regulatory functions in bile acid metabolism and intestinal barrier repair. In the present study, these genera exhibited significant negative correlations with hepatic choline and positive correlations with phosphocholine, indicating their crucial role in promoting choline metabolism and TG export. Emerging evidence indicates that the human gut microbiota modulates the metabolism of L‐tyrosine and L‐phenylalanine (Dodd et al. 2017). Notably, increased abundance of *Bifidobacterium* has been associated with elevated systemic L‐phenylalanine levels. Importantly, L‐phenylalanine exhibits pro‐inflammatory activity, suggesting its potential role in microbiota‐host inflammatory interactions (Yang et al. 2023). The genera *Alistipes*, *Colidextribacter*, *Bifidobacterium*, and *Faecalibacterium* demonstrated significant negative correlations with hepatic L‐tyrosine and L‐phenylalanine levels. This suggests these microbial taxa are critically involved in RRFJ‐mediated reduction of L‐phenylalanine accumulation in HFD‐fed mouse livers, thereby attenuating hepatic inflammation. These findings indicate that RRFJ intervention modulates the gut microbiota of HFD mice, playing a pivotal role in regulating hepatic levels of endogenous metabolites including both lipids and amino acids. The strong association between gut microbiota and hepatic metabolites further supports that RRFJ exerts protective effects in hyperlipidemic mice by restoring gut microbial homeostasis and ameliorating hepatic metabolic disorders through the gut‐liver axis.

**FIGURE 8:**
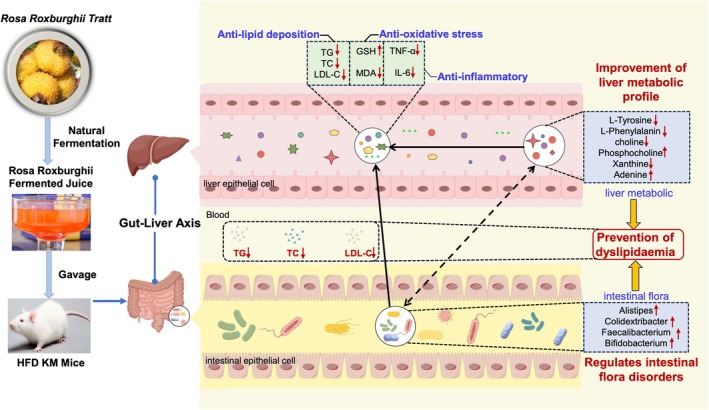
Schematic representation of the mechanism of RRFJ in preventing HFD‐induced dyslipidemia in mice. (By Figdraw).

In recent years, there has been growing consumer interest in natural, health‐promoting, and chronic disease‐preventing medicinal foods Accumulating evidence indicates that many herbal medicines, including ethnomedicinal plants with dual food‐medicine properties, exert bioactive effects through biotransformation by the gut microbiota (Cui et al. 2024). Phytoactive compounds in functional foods mirror traditional Chinese medicine's polypharmacology, this paradigm shift toward dietary‐delivered network pharmacology underscores the transformative potential of TCM‐inspired nutraceuticals (Meng et al. 2025). Aligning with existing evidence on nutraceutical approaches for hyperlipidemia, RRFJ exhibits tripartite therapeutic efficacy by simultaneously ameliorating dyslipidemia, oxidative damage, and low‐grade inflammation. This tridirectional modulation is achieved through microbiota‐dependent mechanisms involving: preservation of intestinal ecological balance, and reprogramming of hepatobiliary metabolic networks via the gut‐liver crosstalk (Fan et al. 2023; Li, Deng, et al. 2022; Pang et al. 2022). While our integrated omics approach has decoded RRFJ's microbiota‐targeting mechanisms, the translational significance warrants confirmation in diet‐controlled trials with microbiota stratification. Clinically, implementation requires precision nutrition strategies that synchronize: habitual dietary patterns, enterotype‐specific microbial signatures, and host metabolic phenotypes (Figure 8).

## 5 Conclusions

In this study, it was discovered that RRFJ (Red Raspberry Fruit Juice) effectively prevented weight gain, high levels of cholesterol, triglycerides, and LDL cholesterol in the blood, as well as liver lipid accumulation caused by a HFD in mice. RRFJ also reduced oxidative stress and inflammation levels. Furthermore, RRFJ improved the disrupted gut microbiota structure caused by the HFD, particularly by decreasing the abundance of certain beneficial bacteria. By analyzing liver metabolites, it was observed that RRFJ intervention led to significant improvements in metabolic profiles, suggesting a role in preventing elevated blood lipids by regulating metabolites such as L‐Tyrosine and L‐Phenylalanine. Pathway analysis indicated that RRFJ influenced metabolic pathways related to lipid deposition. The correlation between liver metabolites and gut flora supports the idea that RRFJ may prevent dyslipidemia through the gut‐liver axis. Further research is necessary to confirm the specific mechanism by which RRFJ intervention effectively prevented dyslipidemia induced by a HFD through the gut‐liver axis. However, based on our current findings, RRFJ could serve as a novel dietary and therapeutic approach to combat dyslipidemia. We recommend exploring and maximizing the potential benefits of *Rosa roxburghii* fermented juice in preventing dyslipidemia and related metabolic disorders.

## Author Contributions

**Duo Meng:** conceptualization (equal), investigation (equal), software (equal), writing – original draft (equal), writing – review and editing (equal). **Pengjiao Wang:** conceptualization (equal), formal analysis (equal), resources (equal), writing – review and editing (equal). **Shuo Zhang:** formal analysis (equal), investigation (equal). **Zhiyu Chen:** data curation (equal), validation (equal). **Chencen Lai:** conceptualization (equal), data curation (equal). **Xinxin Yi:** data curation (equal), software (equal). **Xuncai Huang:** funding acquisition (equal), resources (equal). **Haoxiang Yu:** visualization (equal). **Min Zhang:** conceptualization (equal), project administration (equal), supervision (equal), writing – review and editing (equal). **Xiuli Gao:** conceptualization (equal), funding acquisition (equal), project administration (equal), supervision (equal), writing – review and editing (equal).

## Conflicts of Interest

The authors declare no conflicts of interest.

## Data Availability

Data is contained within the article.
